# Ex vivo anti-malarial drug susceptibility of *Plasmodium falciparum* isolates from pregnant women in an area of highly seasonal transmission in Burkina Faso

**DOI:** 10.1186/s12936-015-0769-1

**Published:** 2015-06-20

**Authors:** Marc C Tahita, Halidou Tinto, Sibiri Yarga, Adama Kazienga, Maminata Traore/Coulibaly, Innocent Valea, Chantal Van Overmeir, Anna Rosanas-Urgell, Jean-Bosco Ouedraogo, Robert T Guiguemde, Jean-Pierre van Geertruyden, Annette Erhart, Umberto D’Alessandro

**Affiliations:** Institut de Recherche en Sciences de la Santé/Direction Régionale de l’Ouest (IRSS/DRO), Bobo-Dioulasso, Burkina Faso; Clinical Research Unit of Nanoro (IRSS-CRUN), Nanoro, Burkina Faso; Unité de Recherche sur le Paludisme et Maladies Tropicales Négligées, Centre Muraz, Bobo-Dioulasso, Burkina Faso; Malariology Unit, Institute of Tropical Medicine (ITM), Antwerp, Belgium; Institut Supérieur des Sciences de la Santé (INSSA), Bobo-Dioulasso, Burkina Faso; International Health Unit, University of Antwerp, Antwerp, Belgium; Medical Research Council Unit, Fajara, The Gambia; London School of Hygiene and Tropical Medicine, London, UK

## Abstract

**Background:**

Ex vivo assays are usually carried out on parasite isolates collected from patients with uncomplicated *Plasmodium falciparum* malaria, from which pregnant women are usually excluded as they are often asymptomatic and with relatively low parasite densities. Nevertheless, *P. falciparum* parasites infecting pregnant women selectively sequester in the placenta and may have a different drug sensitivity profile compared to those infecting other patients. The drug sensitivity profile of *P. falciparum* isolates from infected pregnant women recruited in a treatment efficacy trial conducted in Burkina Faso was determined in an ex vivo study.

**Methods:**

The study was conducted between October 2010 and December 2012. *Plasmodium falciparum* isolates were collected before treatment and at the time of any recurrent infection whose parasite density was at least 100/µl. A histidine-rich protein-2 assay was used to assess their susceptibility to a panel of seven anti-malarial drugs. The concentration of anti-malarial drug inhibiting 50% of the parasite maturation to schizonts (IC_50_) for each drug was determined with the IC Estimator version 1.2.

**Results:**

The prevalence of resistant isolates was 23.5% for chloroquine, 9.2% for mefloquine, 8.0% for monodesethylamodiaquine, and 4.4% for quinine. Dihydroartemisinin, mefloquine, lumefantrine, and monodesethylamodiaquine had the lowest mean IC_50_ ranging between 1.1 and 1.5 nM respectively. The geometric mean IC_50_ of the tested drugs did not differ between chloroquine-sensitive and resistant parasites, with the exception of quinine, for which the IC_50_ was higher for chloroquine-resistant isolates. The pairwise comparison between the IC_50_ of the tested drugs showed a positive and significant correlation between dihydroartemisinin and both mefloquine and chloroquine, between chloroquine and lumefantrine and between monodesethylamodiaquine and mefloquine.

**Conclusion:**

These ex vivo results suggest that treatment with the currently available artemisinin-based combinations is efficacious for the treatment of malaria in pregnancy in Burkina Faso.

**Trial registration:**

ClinicalTrials.gov ID: NCT00852423

**Electronic supplementary material:**

The online version of this article (doi:10.1186/s12936-015-0769-1) contains supplementary material, which is available to authorized users.

## Background

Artemisinin-based combination treatment (ACT) is recommended by the World Health Organization (WHO) for the treatment of uncomplicated falciparum malaria [1]. The combinations comprise a fast-acting artemisinin derivative that rapidly reduces the parasite biomass and gametocyte carriage [2, 3], and a slower-acting partner drugs that clears the remaining parasites and provides post-treatment prophylaxis whose duration depends on its pharmacokinetic properties [4–6]. The rationale for combining anti-malarials with different mechanisms of action is to prevent the development of resistance or at least slow down its onset [1]. Nevertheless, artemisinin-resistant *Plasmodium falciparum* has emerged in western Cambodia and the bordering regions with Thailand, the hotspot of multidrug-resistant parasites [7–10], and is now reported in five countries of the Greater Mekong Subregion, i.e., Cambodia, Thailand, Myanmar, Vietnam, and Laos [11]. Artemisinin-resistant parasites could either spread to other regions and continents or emerge independently in areas of extensive artemisinin use [12]. Therefore, it is important to monitor ACT efficacy and *P. falciparum* sensitivity to different anti-malarials in endemic countries in order to timely update treatment guidelines [13]. This can be done by using different methods, including ex vivo assays that can provide useful information on the susceptibility of the local parasite population to different anti-malarial drugs.

Burkina Faso changed its treatment policy in 2005, from chloroquine monotherapy to ACT with either artemether-lumefantrine (AL) or amodiaquine–artesunate (AS–AQ). Since the implementation of the new policy in 2006, several trials have shown high efficacy of the recommended treatments [14–16], while only one ex vivo assay has been carried out [17]. Ex vivo assays are usually done on parasite isolates from patients with uncomplicated falciparum malaria, mostly children in sub-Saharan Africa. Malaria-infected pregnant women, who represent an important group at risk, are usually not included in these assays as they are often asymptomatic and with relatively low parasite densities [18, 19]. However, *P. falciparum* parasites infecting pregnant women can selectively accumulate in the placenta and represent a distinct sub-set of parasites expressing a unique *P. falciparum* erythrocyte membrane protein 1 (PfEMP1) that binds to the host-receptor chondroitin sulphate A (CSA). These PfEMP1s are structurally, antigenically and functionally distinct from those expressed by parasites that infect children and non-pregnant women [20]. In addition, some studies concluded that parasites seen on the blood smear of a pregnant woman may be CSA-binding parasites derived from the placenta, may be CD36-binding parasites circulating outside the placenta, or may be a mixture of the two subpopulations [21]. It was then hypothesized that such parasites harvested intravenously could have a different sensitivity profile compared to those from children and non-pregnant women. Therefore, ex vivo assays were carried out on isolates collected from pregnant women attending antenatal clinics and diagnosed with malaria in the Burkina Faso study site of a larger multicentre trial.

## Methods

### Study area

The study was carried out at the Clinical Research Unit of Nanoro (CRUN), located at about 85 km from the capital city of Ouagadougou. Malaria is hyperendemic and highly seasonal, occurring between July and December, corresponding to the rainy season. The entomological inoculation rate (EIR) is estimated at 50–60 infective bites/person/year (Diabate A, pers comm). Malaria is the major reason for attending health facilities, with *P. falciparum* being responsible for more than 90% of the infections [22].

### Study design

This study was part of a multicentre (Burkina Faso, Ghana, Malawi, and Zambia) trial (ClinicalTrials.gov ID: NCT00852423) [23] investigating the efficacy and safety of four anti-malarial treatments, namely dihydroartemisinin–piperaquine (DHA–PQ), mefloquine-artesunate (MQAS), AS–AQ and AL in falciparum malaria-infected pregnant women. In Burkina Faso, an ex vivo study on the drug sensitivity of isolates from pregnant women was nested into the trial. Pregnant women were included in the trial if they fulfilled the following inclusion criteria: gestation ≥16 weeks, *P. falciparum* mono-infection at any density with or without symptoms, haemoglobin ≥7 g/dl, residence within the health facility catchment’s area, and willingness to deliver at the health facility. For the ex vivo study the inclusion was limited to women with a parasite density of at least 100/µl. The study protocol was approved by the respective ethical review boards (the Ethics Committee of the University Hospital of Antwerp, Institutional Ethic Committee of Centre Muraz and the National Ethic Committee) and written informed consent was obtained from all study participants.

### Collection of field isolates

*Plasmodium falciparum* field isolates were collected at day 0 (before treatment) and at any day of recurrent infection during a 63-day in vivo drug efficacy follow-up. A volume of 3–4 ml of venous blood was collected in heparinized tubes and transferred within 24 h to the laboratory for testing. Blood was centrifuged, plasma and buffy coat were removed, and parasitized erythrocytes were washed three times with RPMI 1640 medium (Sigma-Aldrich, St Louis, USA) at 37°C. Field parasite samples with more than 1% parasitaemia were diluted with uninfected erythrocytes (human blood type O+) to avoid any influence of an inoculum effect on assay results. Prior to the study start, successful experiments were conducted with initial parasite densities as low as 0.002%.

### Drug sensitivity testing

Monodesethylamodiaquine (MDAQ), chloroquine diphosphate (CQ), quinine hydrochloride (QN) and mefloquine (MQ) were purchased from Sigma-Aldrich (St Louis, USA). Dihydroartemisinin (DHA) and piperaquine phosphate (PiP) were donated by Sigma-Tau (Rome, Italy) and lumefantrine (LUM) by Novartis Pharma (Basel, Switzerland). The drugs stock solutions were prepared at a concentration of 1 mg/ml in the following solvents: MDAQ and CQ in distilled water, MQ and PiP in lactic acid, LUM in ethanol, QN and DHA in methanol. Multiple wells of a 96-well culture plate were predosed with twofold serial dilutions of each drug at final concentrations that ranged from 12.5 to 3.200 nM for CQ, 6.25–400 nM for MDAQ, 13–3.333 nM for QN, 0.2–64 nM for DHA, 1.2–300.2 nM for LUM, 1.6–100 nM for PiP and 3.2–206.3 nM for MQ. For each sample, 200 µl aliquots of cultured parasites, prepared as described above, were added to each well and incubated at 37°C at 5% CO2 for 72 h. The culture plates were then frozen and stored at −20°C for up to 4 weeks.

Parasite growth inhibition was quantified using an enzyme-linked immunosorbent assay (ELISA) that quantifies parasite histidine-rich protein-2 (HRP-2) [24]. Two commercially available monoclonal antibodies (Immunology Consultants Laboratory, Inc, Newberg, OR, USA) directed against *P. falciparum*-specific HRP2, i.e., MPFM-55A and MPFG-55P, were used for the ELISA. Plates were pre-coated with the first monoclonal antibody MPFM-55A (original concentration from the manufacturer was 7,200 μg/ml) diluted at a concentration of 1.0 μg/ml. In order to obtain complete haemolysis before starting the ELISA, cultured samples were diluted 1:2 in distilled water. One-hundred microliter of each haemolyzed samples were added to the ELISA plate for 1 h then washed three times with the washing solution (PBS + 0.05% Tween 20, Sigma-Aldrich). The second antibody (MPFG-55P) was diluted at a concentration of 0.05 µg/ml and then added (100 µl) for 1 h at room temperature, washed again three times, and incubated with 100 µl of TMB (3,3′_,5,5′-tetramethylbenzidine) chromogen (Sigma-Aldrich, St Louis, USA) in the dark for 5–10 min. After incubation the reaction was stopped with 50 µl of 1 M sulfuric acid. Spectrophotometric analysis was performed with an ELISA plate reader (Multiskan FC, plate reader) at 450 nm.

### Statistical analysis

Inhibitory concentrations (IC_50s_) for anti-malarials were calculated using IC50 Estimator Version 1.2 available online [25]. Data were entered in Excel version 97 and analyzed with Stata version 10.0. Results were expressed as geometric mean IC_50_ (concentration at which 50% of parasite growth was inhibited) with 95% confidence intervals. Pairwise comparisons were done and the Spearman rank-order correlation test used to determine the correlation between IC_50_ values. For all statistical tests, the significance level was set at p ≤ 0.05. The threshold IC_50_ for ex vivo resistance was defined as ≥100 nM for CQ, ≥60 nM for MDAQ, ≥800 nM for QN [26], and ≥30 nM for MQ [27, 28].

## Results

A total of 108 *P. falciparum* isolates were tested and 90 (83.3%) had interpretable results for at least one of the study drugs (Table 1). The overall culture success rate was around 80% and varied little between study drugs (from 76.8 to 83.3 %). Missing results were due to poor ex vivo growth or failure to achieve adequate fit on a log dose–response curve.

**Table 1:** Ex vivo susceptibility of *Plasmodium falciparum* isolates by drug tested

	Culture success rate % (n/N)	Geometric Mean IC50 (nM) [95% CI]	Range		Resistant isolates n (%)
MDAQ	80.6 (87/108)	1.5 [1.0–2.2]	0.1	158.5	7 (8.0)
MQ	80.6 (87/108)	1.1 [0.8–1.7]	0.02	103.7	8 (9.2)
DHA	79.6 (86/108)	1.2 [0.9–1.6]	0.1	10.2	NA*
CQ	78.7 (85/108)	40.1 [28.6–58]	1.2	1060.1	20 (23.5)
LUM	78.7 (85/108)	1.4 [1.0–2.1]	0.1	97.7	NA
QN	83.3 (90/108)	34.2 [24.1–48.5]	1.9	995.5	4 (4.4)
PiP	76.8 (83/108)	5.0 [4.1–6.3]	0.5	47.9	NA

Resistance threshold defined for CQ at 100 nM, QN at 800 nM, MQ at 30 nM and MDAQ at 60 nM.

* *NA* not applicable as the threshold is not defined yet.

CQ (geometric mean IC_50_ = 40.7 nM; 95% CI 28.6–57.9) had the highest rate of resistant isolates (23.5%), followed by MQ (geometric mean IC_50_ = 1.1 nM; 95% CI 0.8–1.7) with 9.2%, MDAQ (geometric mean IC_50_ = 1.5; 95% CI 1.0–2.2) with 8.0%, and then QN (geometric mean IC_50_ = 34.2; 95% CI 24.1–48.5) with 4.4%. DHA, LUM and MDAQ had a low IC_50_ that ranged between 1.1 and 1.5 nM (Table 1). The geometric mean and the resistance cutoff (where available) are shown in Additional file 1: Figure S1. Most drugs were equally active against the CQ-sensitive and CQ-resistant isolates except for QN where the IC_50_ for CQ-resistant isolates was higher than that for CQ-sensitive ones, although the difference did not reach statistical significance (Table 2). There was a positive significant correlation between the sensitivity of DHA and both MQ and CQ, between CQ and LUM and between MDAQ and MQ (Table 3).

**Table 2:** Geometric mean IC_50_ of the different drug tested by chloroquine sensitivity

Drug	Chloroquine-resistant isolates IC_50_ (95 % CI) N = 20	Chloroquine-sensitive isolates IC_50_ (95 % CI) N = 65	*P* value
MDAQ	1.2 (0.6–2.7)	1.4 (0.9–2.2)	0.98
MQ	0.9 (0.4–1.9)	1.2 (0.8–2.0)	0.317
DHA	1.8 (0.9–3.4)	1.1 (0.8–1.5)	0.23
CQ	487.2 (334.9–708.9)	18.9 (15.2–23.7)	<0.001
LUM	2.1 (0.7–5.9)	1.3 (0.8–2.0)	0.18
QN	64.4 (29.1–142.1)	28.8 (19.4–42.7)	0.06
PiP	6.6 (4.3–10.1)	4.7 (3.6–6.1)	0.29

**Table 3:** Pairwise comparison between the IC_50_ of tested drugs

Drug pairs	r^a^	P value
MDAQ–MQ	0.36	<0.001*
MDAQ–DHA	0.13	0.22
MDAQ–CQ	0.13	0.21
MDAQ–LUM	0.14	0.20
MDAQ–QN	0.13	0.20
MDAQ–PiP	−0.037	0.74
MQ–DHA	0.23	0.02*
MQ–CQ	−0.03	0.78
MQ–LUM	0.10	0.35
MQ–QN	0.08	0.43
MQ–PiP	−0.05	0.60
DHA–CQ	0.21	0.04*
DHA–LUM	0.19	0.07
DHA–QN	0.07	0.48
DHA–PiP	0.14	0.20
CQ–LUM	0.21	0.05
CQ–QN	0.27	0.001*
CQ–PiP	0.21	0.05
LUM–QN	0.01	0.90
LUM–PiP	0.17	0.11
QN–PiP	−0.09	0.41

^a^Spearman’s rank-order correlation coefficient (r).

## Discussion

As expected, a substantial proportion of isolates were resistant to CQ while for the other drugs whose sensitivity threshold is known, the prevalence of resistant isolates was <10%. The prevalence of CQ-resistant (CQ-R) isolates in Bobo-Dioulasso, a town situated at several hundreds of kilometres to the southwest of Nanoro, was 50% in 2006 (Lea Bonkian, pers comm) and 42.1% in 2008–2010 [17], a slight decrease that coincided with the change in the national treatment policy. As the latter was implemented in 2006, it may be too early to detect a substantial decrease of CQ-R isolates, although this may happen. Indeed, such a decrease has been observed in other African countries after withdrawing CQ as first-line treatment and hence decreasing the selective pressure on the local parasite population [29–31]. In Nanoro, the prevalence of CQ-R isolates as well as the CQ mean IC_50_ were lower than in Bobo-Dioulasso. However, these may have already been lower in the past and probably reflect the higher drug pressure usually found in urban (Bobo-Dioulasso) compared to rural (Nanoro) areas. To make any conclusion on the trend of CQ resistance, it is necessary to carry out serial ex vivo studies at regular intervals and in the same sites.

The persistence of CQ-R *P. falciparum* isolates may be due to the continued use of CQ by the local population [32], maintaining the drug selective pressure, and/or the cross-resistance to other drugs with similar chemical structure. Indeed, the use of AS–AQ as one of the first-line treatments may contribute to maintain the drug pressure on CQ-R parasites and could explain the high prevalence found in this study. Nevertheless, MDAQ seems to have a good activity on the local parasites, with only seven (8.0%) isolates with IC_50_ above the accepted threshold of resistance, indicating that AS–AQ should still have a reasonable efficacy in pregnant women with malaria. LUM, MQ and PiP are partner drugs in ACT and all of them had a relatively low mean IC_50_, the first two around 1 nM and the latter at 5.0 nM. In addition, DHA’s mean IC_50_ was also low, confirming earlier reports from other parts of Burkina Faso as well as other sub-Saharan African countries [17, 33–35]. This suggests that the efficacy of both AL and AS–AQ should be good as most parasite isolates were sensitive to both components of the two ACT. The prevalence of MQ-resistant isolates was slightly higher than that of MDAQ, indicating that MQAS efficacy in vivo would probably be similar to that of AS–AQ. It is important to point out that the prevalence of *P. falciparum* isolates resistant to MQ was higher in other West African countries where ex vivo studies were carried out [27, 36].

PiP had a higher mean IC_50_ than the other partner drugs in currently available ACT, namely MDAQ, MQ and LUM. Nevertheless, the upper value of its range was lower and the PiP IC_50_ in CQ-R and CQ-S isolates was not significantly different, indicating that although belonging to the same class as CQ and AQ, PiP may be much more efficacious. Therefore, the combination DHA–PiP, although not recommended yet for the treatment of malaria in pregnancy, may be the most promising among currently available ACT. In addition, given PiP’s long elimination half-life, it would have the advantage of providing a long post-treatment prophylaxis period in which the patient could be protected from emerging infections.

QN, together with CQ, had one of the highest mean IC_50_. Oral QN was recommended until recently (2014) in Burkina Faso for the management of uncomplicated malaria during pregnancy, including during the first trimester [37], and it is used as rescue treatment in case of ACT failure. Although just a few isolates were above the resistance threshold, QN IC_50_ was strongly associated to that of CQ and was much higher in CQ-R than CQ sensitive (CQ-S) isolates, indicating some cross-resistance. A similar association was observed in a previous ex vivo study carried out in Burkina Faso in an urban area, although the mean IC_50_ was higher, possibly indicating a higher drug selective pressure [17]. The relative good efficacy of most of the drugs tested in our study is encouraging, as these drugs will rapidly clear the circulating parasites. This is particularly important as peripheral infection confers a five-fold increased risk of placental malaria [38] and can therefore have a markedly negative impact on mothers and babies [39, 40].

## Conclusion

*Plasmodium falciparum* parasites isolated from pregnant women show a drug sensitivity profile comparable to that recently reported from Bobo-Dioulasso, Burkina Faso [17]. The mean IC_50_ values and the prevalence of isolates resistant to drugs for which the threshold is known were generally lower in Nanoro than in Bobo-Dioulasso, possibly due to the lower drug pressure in this rural area. Indeed, it is not possible to ascribe the observed differences to the type of patients from which the isolates were collected, namely pregnant women in Nanoro and children in Bobo-Dioulasso. Ex vivo results indicate that all currently available ACT would probably have good efficacy among pregnant women with malaria. Nonetheless, the therapeutic response is not only dependent on the parasite susceptibility but also on the dose given, the drug disposition and metabolism. Pregnancy is associated with physiological changes that may alter the pharmacokinetic of treatments administered and hence influence the therapeutic response [41]. In vivo treatment efficacy will be provided by the trial in which this ex vivo study is nested. The drug sensitivity profile of the parasite population circulating in Nanoro represents the baseline on which to monitor the evolution of the drug sensitivity profile of the local parasite population.

## Additional file

Additional file 1:
**Figure S1.** Scatter of the IC50 values with geometric mean and the resistance cutoff (where available) of each drug. In order to better visualize and understand the data, a figure showing the scatter of the IC50 values with geometric mean and the resistance cutoff (where available) of each drug is proposed.
