# Supplementary material for: Ex vivo anti-malarial drug susceptibility of Plasmodium falciparum isolates from pregnant women in an area of highly seasonal transmission in Burkina Faso
Source: Malar J. 2015 Jun 20;14:251. doi: 10.1186/s12936-015-0769-1 (PMC4474342; doi:10.1186/s12936-015-0769-1)

Additional file: Figure S1 Scatter of the IC50 values with geometric mean and the resistance cutoff (where available) of each drug

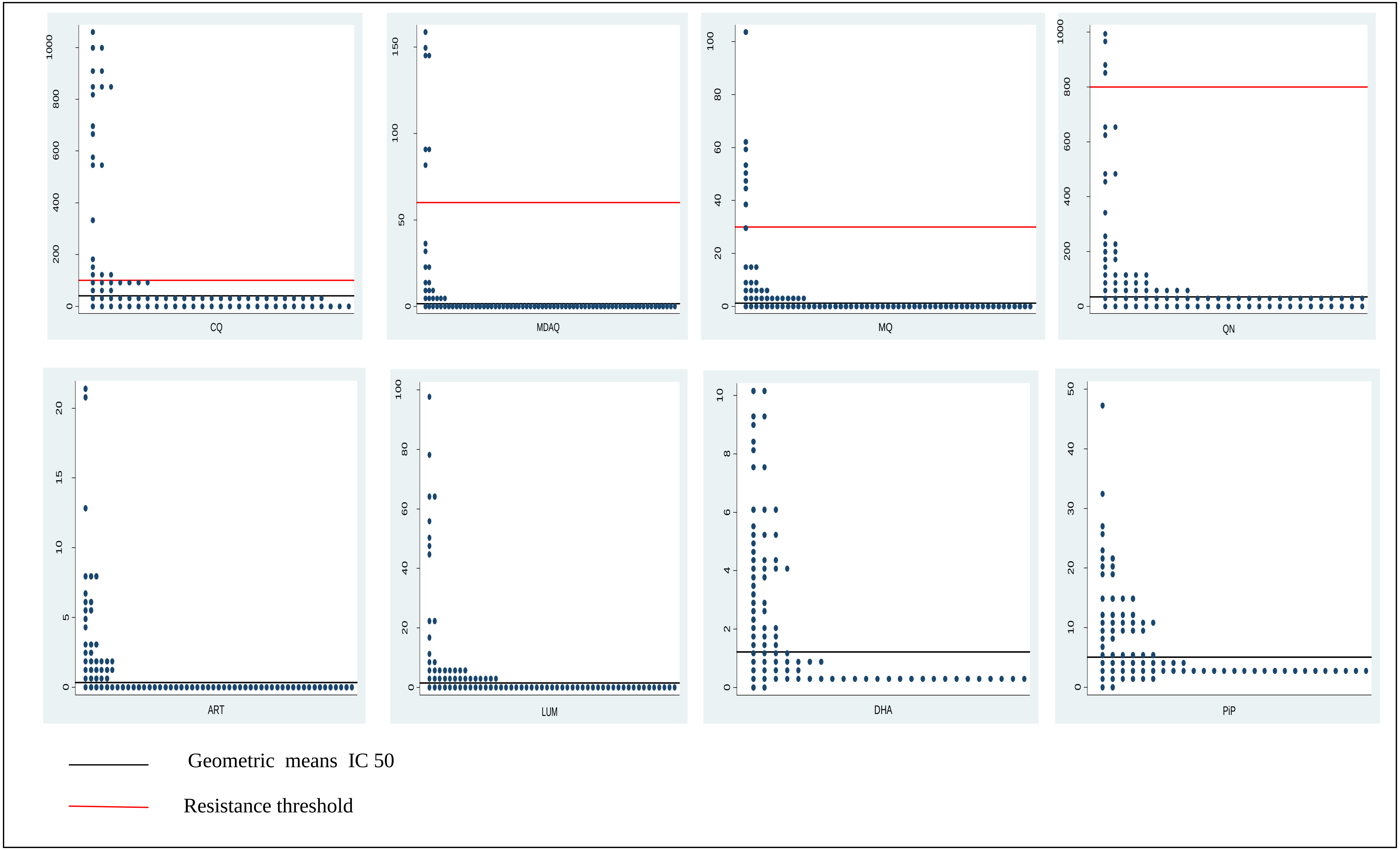

Supplement: Additional file 1: — Figure S1. Scatter of the IC50 values with geometric mean and the resistance cutoff (where available) of each drug. In order to better visualize and understand the data, a figure showing the scatter of the IC50 values with geometric mean and the resistance cutoff (where available) of each drug is proposed. [file 12936_2015_769_MOESM1_ESM.pdf]
